# Reflex control of the spine and posture: a review of the literature from a chiropractic perspective

**DOI:** 10.1186/1746-1340-13-16

**Published:** 2005-08-09

**Authors:** Mark W Morningstar, Burl R Pettibon, Heidi Schlappi, Mark Schlappi, Trevor V Ireland

**Affiliations:** 1 Director of Research; The Pettibon Institute, 3416-A 57 St Ct NW Gig Harbor, WA 98335, USA; Private practice of chiropractic, 10683 S Saginaw St, Suite B, Grand Blanc, MI 48439, USA; 2 Executive Director; The Pettibon Institute, 3416-A 57 St Ct NW Gig Harbor, WA 98335, USA; 3 Doctor of Chiropractic Candidate; Palmer College of Chiropractic. 1000 Brady St Davenport, IA 52803, USA; 4 Board of Trustees; Palmer College of Chiropractic. 1000 Brady St Davenport, IA 52803, USA

**Keywords:** Cervical spine, Posture, Reflex

## Abstract

**Objective:**

This review details the anatomy and interactions of the postural and somatosensory reflexes. We attempt to identify the important role the nervous system plays in maintaining reflex control of the spine and posture. We also review, illustrate, and discuss how the human vertebral column develops, functions, and adapts to Earth's gravity in an upright position. We identify functional characteristics of the postural reflexes by reporting previous observations of subjects during periods of microgravity or weightlessness.

**Background:**

Historically, chiropractic has centered around the concept that the nervous system controls and regulates all other bodily systems; and that disruption to normal nervous system function can contribute to a wide variety of common ailments. Surprisingly, the chiropractic literature has paid relatively little attention to the importance of neurological regulation of static upright human posture. With so much information available on how posture may affect health and function, we felt it important to review the neuroanatomical structures and pathways responsible for maintaining the spine and posture. Maintenance of static upright posture is regulated by the nervous system through the various postural reflexes. Hence, from a chiropractic standpoint, it is clinically beneficial to understand how the individual postural reflexes work, as it may explain some of the clinical presentations seen in chiropractic practice.

**Method:**

We performed a manual search for available relevant textbooks, and a computer search of the MEDLINE, MANTIS, and Index to Chiropractic Literature databases from 1970 to present, using the following key words and phrases: "*posture*," "*ocular*," "*vestibular*," "*cervical facet joint*," "*afferent*," "*vestibulocollic*," "*cervicocollic*," "*postural reflexes*," "*spaceflight*," "*microgravity*," "*weightlessness*," "*gravity*," "*posture*," and "*postural*." Studies were selected if they specifically tested any or all of the postural reflexes either in Earth's gravity or in microgravitational environments. Studies testing the function of each postural component, as well as those discussing postural reflex interactions, were also included in this review.

**Discussion:**

It is quite apparent from the indexed literature we searched that posture is largely maintained by reflexive, involuntary control. While reflexive components for postural control are found in skin and joint receptors, somatic graviceptors, and baroreceptors throughout the body, much of the reflexive postural control mechanisms are housed, or occur, within the head and neck region primarily. We suggest that the postural reflexes may function in a hierarchical fashion. This hierarchy may well be based on the gravity-dependent or gravity-independent nature of each postural reflex. Some or all of these postural reflexes may contribute to the development of a postural body scheme, a conceptual internal representation of the external environment under normal gravity. This model may be the framework through which the postural reflexes anticipate and adapt to new gravitational environments.

**Conclusion:**

Visual and vestibular input, as well as joint and soft tissue mechanoreceptors, are major players in the regulation of static upright posture. Each of these input sources detects and responds to specific types of postural stimulus and perturbations, and each region has specific pathways by which it communicates with other postural reflexes, as well as higher central nervous system structures. This review of the postural reflex structures and mechanisms adds to the growing body of posture rehabilitation literature relating specifically to chiropractic treatment. Chiropractic interest in these reflexes may enhance the ability of chiropractic physicians to treat and correct global spine and posture disorders. With the knowledge and understanding of these postural reflexes, chiropractors can evaluate spinal configurations not only from a segmental perspective, but can also determine how spinal dysfunction may be the ultimate consequence of maintaining an upright posture in the presence of other postural deficits. These perspectives need to be explored in more detail.

## Background

Historically, chiropractic has centered around the concept that the nervous system controls and coordinates all other systems within the human body [1,2]. Recent evidence has provided insight into the mechanisms responsible for this neurological governance of other body systems [3-9]. Perhaps the most important relationship from a chiropractic perspective, however, is that between the nervous and musculoskeletal systems. Specifically, many chiropractors believe that "subluxations" of the vertebral column somehow compromise the integrity and function of the nervous system, which may ultimately affect health and vitality [10]. However, to date, research attempting to identify the exact parameters of the chiropractic subluxation remains tenuous [11,12].

More recently, certain authors [13,14] have discussed an alternative concept of neurological dysfunction. Two virtually synonymous concepts, dysafferentation [13] and the wind-up phenomenon [14], are based on the premise that neurological dysfunction is caused by a constant barrage of afferent input into the nervous system, causing a hypersensitive state within the neuronal receptor pool. These receptor pools, made largely of interneurons, allow sensory input to be conveyed to higher spinal and cortical centers, while simultaneously providing the means for spinal reflexive control of various functions [13,15]. Neurologic dysfunction caused by afferent stimulation may be related to certain types of headache [14], joint dysfunction, and muscular restriction [13].

The chiropractic interest in static global spinal structure and its correction is growing [16-27]. Most of this research has only surfaced within the last 10 years. Much of this research is focused upon the inherent biomechanics of the vertebral column. Research in the areas of spinal modeling [16,17,24,26,27] and posture analysis [21] have attempted to provide a clinically valid outcome measure for the treatment of posture-related symptoms and pathologies. For example, Wiegand et al [27] demonstrated a correlation between certain cervical spinal configurations and the presence of pathology. Harrison et al [17,24,26] reported average ranges of the sagittal spine curves for 3 sets of asymptomatic populations. This type of biomechanical modeling is important for developing parameters by which outcome assessments can be created and implemented. Unfortunately, spinal modeling cannot account for the host of mechanisms and precipitating factors that promote the divergence of the spine away from these established biomechanical models. However, these concepts and models do not account for, or acknowledge, the importance of the neurological, reflexive control of posture. Rather than simply identifying that a given patient does not fit into a normal spinal model, further investigation into why that particular patient does not fit is perhaps more important in terms of developing patient management strategies. This is important not only for understanding why abnormal spinal configurations occur, but to also discuss the potential to recruit these same neurological pathways to aid in the correction of spinal or postural abnormalities.

Postural reflexes can be subcategorized as the following: visual righting reflexes, labyrinthine righting reflexes, neck righting reflexes, body on head righting reflexes, and body on body righting reflexes [28]. Although some of the reflexes and neuroanatomy have been defined and illustrated separately, these collective reflexes and their interactions have not been examined from a chiropractic perspective. Since conservative postural treatment is becoming increasingly investigated, knowledge of the postural reflexes will only aid the practitioner in providing treatment consistent with foundational postural neurophysiology. In our review, we will illustrate the mechanisms by which the nervous system controls and coordinates posture, with special emphasis placed on how the nervous system adapts to specific external environmental factors. This review will detail the neurological control of posture, specifically the afferent regulation of posture. We will illustrate the neuroanatomy involved in afferent postural control, giving most attention to those reflexes associated with the cervical spine and special senses. We also discuss the interactions between the various afferent structures and their postural effects.

The primary purpose of the postural reflexes is to maintain a constant posture in relation to a dynamic external environment. This review will discuss the main external environmental parameter by which these reflexes maintain and adapt postural control: gravity. Because earth's gravitational field is a constant, the postural reflexes develop and react to this constant. From the moment an infant learns to first hold its head up through the time the child begins to walk upright, these postural reflexes are essentially supervising spinal structural and functional development in direct response to the constant force of gravity. To allow for a balance of strength and flexibility, the spine develops natural sagittal curves that provide functional lever arms for muscular attachment and efficient movement. Again, all of this is achieved using the constant of gravity as the main reference point, and the postural reflexes serve as the neuromotor impetus for this adaptive response.

This review will also detail the mechanisms that cause the reactive musculoskeletal changes in response to sudden changes in the external environment. Primarily, we will illustrate and compare the effects of gravitational changes upon the cervical spine postural reflexes and resultant postural adaptations. Specifically, details of postural adaptation, musculoskeletal morphological changes, and clinical symptoms in microgravitational environments will be outlined and discussed.

## Methods

Starting from the year 1970, we searched the MEDLINE database using the following key words and phrases: "*posture*," "*ocular*," "*vestibular*," "*cervical facet joint*," "*afferent*," "*vestibulocollic*," "*cervicocollic*," and "*postural reflexes*," "*spaceflight*," "*microgravity*," "*weightlessness*," "*gravity*," and "*postural*." Searches of the MANTIS database and the *Index to Chiropractic Literature *using the same key word were also performed. Nearly all of the articles relating to our review were also found on MEDLINE. A hand search of our personal libraries was also conducted, retrieving textbooks pertaining to this topic. For purposes of this review, we included original research articles, review papers, case series, or textbook chapters outlining the anatomy, physiology, evaluation, or pathophysiology and interaction of vision, the vestibular system, the vertebral column, or a combination of these. This review was organized so that a brief review of each structure could be discussed both individually and collectively. Although these databases house a vast multitude of articles on posture, only those specifically pertaining to neurological or neuromuscular control were included.

### Visual Input

The visual pathway consists of the following parts: the optic nerve, optic chiasm, and the optic tracts which project to three subcortical areas known as the pretectum, the superior colliculus, and the lateral geniculate body. Information relayed by this pathway ascends from the optic nerve ultimately to the lateral geniculate body, with axons projecting to the primary visual cortex [29]. The primary visual cortex is located on the medial surface of the occipital lobe in the walls of the calcarine sulcus. [29]

The visual field and pathway are important regulators of postural control. Visual input for postural control helps to fixate the position of the head and upper trunk in space, primarily so that the center of mass of the trunk maintains balance over the well-defined limits of foot support [24]. Many studies have shown the destabilizing effects on postural regulation when the visual field is altered due to injury, disease, or congenital abnormality [31-38]. Guerraz et al [34] studied 21 patients diagnosed with visual vertigo. They found that subjecting these patients to disorienting visual environments markedly reduced postural control. Catanzariti et al [31] identified a correlation between the severity of postural deformity in scoliosis patients who present with visual disorders.

It is well known that vision has a major role in the regulation of upright posture, particularly by maintaining head position in space. Alterations in head posture may develop secondarily to visual changes. For example, Havertape and Cruz [35] showed how the addition of eyeglasses changed the head position in 5 patients with a chin-down posture as a result of high hyperopia. Likewise, Willford et al [39] showed that people who wear prescription multifocal lenses tend to exaggerate a forward head posture to utilize the proper area of the lense, depending upon the functional needs of the moment. This has important implications for posture rehabilitation and will be discussed in detail in this review. In a study of 125 patients with congenital nystagmus, Stevens and Hertle [38] found that those patients who assumed a compensatory abnormal head posture achieved better visual acuity than those who failed to adapt to the presence of the nystagmus. In 5 patients with unilateral vision loss due to cyclotropia or monocular nystagmus, Nucci and Rosenbaum [36] found that a compensatory head tilt or rotation could be reduced by surgical correction of the ocular disorder. Pyykko et al [37] conducted a study on 10 patients with Usher's syndrome and 10 patients with blindness. All 20 patients displayed a statistically significantly higher postural sway than the control group. It is noteworthy to point out that visual information relayed to higher centers is based upon relative information. Although postural control is highly dependent upon visual status, higher cortical functions are necessary to differentiate between a fixed person within a moving environment, or a moving person within a fixed environment. Buchanan et al [30] demonstrated how the central nervous system might actively suppress visual information that is inconsistent with afferent postural control input from other sources, such as the somatosensory system.

While vision is an important part of postural control, the information it relays to higher cortical areas remains based on relative perception. Postural corrections initiated by the visual system are made in the direction of visual stimulus [40]. Afferent stimulus provided by the visual field can include either movement of the environment around the person, or movement of the person in the environment [33]. As Guerraz et al [33] and DiZio et al [41] have pointed out, small changes in the visual environment can alter visually based posture control, such as darkness or changes below the conscious threshold. However, visual control of posture in real time does not receive much contribution from higher-level processes [42]. As infants learn to assume a sitting position, much of this postural development relies upon input from the visual environment. As the child repeats a sitting task, a visuomotor coordination develops, and becomes extremely sensitive to visual variables. As the child learns to stand and walk, however, the visual input must now coordinate with other postural control mechanisms, such as joint mechanoreceptors of the hips, knees, and ankles [42].

Aside from the visual field itself providing an important source of postural control, proprioceptive information may also be relayed from the extraocular muscles themselves. Buttner-Ennever and Horn [43] describe a 'dual control' system where two distinct pathways are responsible for afferent input into the oculomotor nuclei. One pathway serves to generate eye rotations, while the second pathway provides sensory information regarding eye alignment and stabilization [44]. This is an important part of the visual postural control pathway, as this pathway may compensate for visual deprivation such as in darkness. This ocular proprioceptive pathway passes through the optic tract nucleus to the rostral portion of the superior colliculus [45,46].

The superior colliculus is known for its essential role in head and eye orientation and coordination [47,48]. It serves as an important integration center for the extraocular proprioceptive pathway as well as the spinal trigeminal nucleus. The superior colliculus also has an extensive reciprocal feedback pathway with the reticular formation, which may also play a role in extraocular proprioception [49]

To further summarize the importance of vision in postural control, Buchanan et al [30] concluded that fixing the head and trunk in space achieves three major functional tasks: 1) it stabilizes the visual field for gaze stabilization, 2) it stabilizes the center of mass of the head and trunk within feet support, and 3) it minimizes the external stress acting upon the head and trunk. Because Buchanan et al [30] showed how visual deprivation destabilizes head and trunk position, this provides evidence that control of the head and trunk is assumed in a top-down mode. This organization may have clinical value when designing treatments to correct abnormal posture.

### Vestibular Input

The vestibular system is an integral component in many of the postural reflexes, especially those that are responsible for upright human posture. The primary function of the vestibular apparatus is to provide sensory input about sustained postural stimulation [50]. The vestibular apparatus is composed of the utricle, saccule, and semicircular canals. Each of these organs is designed to detect specific types of motion. The utricle and saccule detect linear accelerations of the head in space. Since gravity exerts a constant vertical acceleration on the head and body, the utricle and saccule provide postural input on head position relative to gravity [50]. The semicircular canals relay afferent input about angular acceleration, such as head rotation. Buttner-Ennever [51] detailed the many connections from the utricle and saccule to the brainstem and cerebellum. The utricle detects changes in head position relative to gravity, such as a simple tilting of the head. The saccule, on the other hand, contributes a partial role in maintaining head position relative to the visual field.

Afferent information is collected and transmitted to higher levels by the vestibular nerve. The vestibular nerve carries afferent input from both the utricle and saccule, where it is transmitted to the lateral vestibular nucleus. Vestibular nuclei receive sensory input from the vestibular nerve as well as information from the cerebellum and the optic tract. Axons from the vestibular nuclei project to the thalamus, superior colliculus, reticular formation, cerebellar flocculus, and lower vestibulospinal nuclei. Of the vestibular nuclei, the lateral vestibular nucleus, or Deiter's nucleus, is perhaps one of the most important nuclei related to postural reflexes, through its projections to the vestibulospinal tract. The vestibulospinal tract and reflex will be discussed later in this review.

Previous experiments have illustrated the effects of vestibular loss on overall postural control [52-54]. Horak et al [53] compared 6 subjects with bilateral vestibular loss to 6 age and sex-matched controls. After subjecting each group to various postural tasks, they found that the experimental group showed increased head and trunk displacements compared to matched controls. In a similar study by Creath et al [52], they found that subjects with bilateral vestibular loss demonstrated a higher center-of-mass variability. However, this variability was reduced with the addition of light-touch fingertip contact. This suggests that despite vestibular deficits, postural control can be maintained by other afferent postural input. Schweigart et al [55] described how subjects with vestibular degradation could compensate with neck proprioception in instances of static postural stance, although postural control is significantly altered when the subject is moving.

### Visual and Vestibular Interactions

While the visual and vestibular systems are individually two of the most important postural reflexes, it's their constant interaction that makes the control of upright posture possible, especially when considering their combined role in the reflex modulation of muscular tone through various groups of postural muscles. The visual and vestibular systems interact primarily through a series of reflexes and tracts, namely the vestibulo-ocular reflex [56-62], the vestibulospinal tract [50,63], and the dorsal and ventral spinocerebellar tracts [64-69].

The vestibulo-ocular reflex serves to orient the visual field by creating certain eye movements that compensate for head rotations [59,62] or accelerations [61]. The vestibulo-ocular reflex may be subdivided into three major components: 1) the rotational vestibulo-ocular reflex, which detects head rotation through the semicircular canals, 2) the translational vestibulo-ocular reflex, which detects linear acceleration of the head via the utricle and saccule, and 3) the ocular counter-rolling response, or optokinetic reflex, which adapts eye position during head tilting and rotation [50]. Through detection of head orientation in space, the vestibular apparatus transmits this information to the vestibular nuclei, where connections with the visual field aid in the correction and coordination of head and body posture via the vestibulo-ocular reflex [56]. The cerebellar flocculus may ultimately be responsible for integrating and executing the efferent corrections of the vestibulo-ocular reflex. Previous research has shown that resection of the cerebellar flocculus permanently prevents vestibulo-ocular reflex response, providing evidence for its direct involvement [58].

The medial and lateral vestibulospinal tracts may be viewed as the efferent equivalents of the vestibulo-ocular reflex, modulating motor neuron activity regarding the axial and appendicular muscles respectively so that rapid postural adaptations can take place. The cerebellum, where afferent information is collected from the visual field, the vestibular nerve, and the cervical mechanoreceptors, and is interpreted for generation of reactive postural corrections, modulates these tracts. Originating in the lateral and medial vestibular nuclei [66], these tracts allow the trunk and extremities to compensate for changes in head position. Reflexive responses from the vestibulospinal tracts help correct sudden perturbations in static upright posture. While the visual input may be more important in constant postural adaptation, the vestibular apparatus, via the vestibulospinal tracts, is much quicker to respond to early or slight postural disruptions, allowing for a faster response from the skeletal postural muscles [50].

Normal visual-vestibular interaction also incorporates afferent input from the dorsal and ventral spinocerebellar tracts. These tracts transmit sensory signals to the cerebellum regarding position sense of the lower extremity [65], primarily through joint, skin, muscle spindle, and golgi tendon organ afferents [64]. These tracts not only provide information relating the position of each lower extremity, but also in coordinating both lower extremities for combined postural tasks such as locomotion [70,71]. The spinocerebellar tracts arise from spinal interneurons within the gray matter between the first thoracic and the second lumbar segments, known as Clarke's nucleus [66]. These interneurons, in turn, communicate with both the afferent and efferent pathways of lower extremity neural control, via spinal reflexes. The clinical importance of this will be discussed in greater detail.

### Cervical Mechanoreceptors

The cervical spine is a virtual warehouse of postural afferent input and integration. Several anatomic structures in this region, including the facet joint and capsule [72-78], spinal ligaments [71], and proprioceptive input from the cervical musculature [70,79,80] are collectively responsible for maintaining an orthogonal head on neck position. In order to understand how these various structures participate in postural regulation, observation of postural control changes in the presence of functional deficits provides evidence of their individual contributions.

The cervical facet joint houses a variety of mechanoreceptors responsible for providing afferent postural input to higher neurological pathways, including connections with the trochlear, abducens, spinal trigeminal, central and lateral cervical, and vestibular nuclei [81-87], as well as the cerebellar flocculus and vermis [83,84,88]. Several types of cervical facet mechanoreceptors have been identified [85,86]. Cervical facet joint mechanoreceptors may be dominant over the vestibular apparatus in regards to the maintenance of static posture [89,90]. For example, when the cervical facet joints are experimentally immobilized in the presence of vestibular dysfunction, postural instability becomes apparent [91]. However, postural stability is restored when the facet joints are mobilized. The facet joint has been the focus of several recent studies regarding whiplash type injuries. Specifically, the facet joints and capsules have been identified as a probable cause in chronic whiplash symptoms in the absence of obvious radiographic injury. A significant number of free nerve endings and lamellated corpuscles were found within the facet joint capsules [75]. These structures are important in the rapid adaptation of changes in cervical spine position. In a study of 105 patients with chronic whiplash symptoms, Treleaven et al [76] found that whiplash patients could not consistently reproduce a natural resting head position when compared to matched controls. Incidentally, Rubin et al [92] report that people with whiplash symptoms have a higher likelihood of suffering from balance failures. Since cervical facet joints contribute to postural orientation, injury to these joints may produce postural symptoms like vertigo and dizziness [76].

In addition to the facet joints, the paraspinal ligaments, such as the posterior longitudinal ligament, also contribute an extensive amount of sensory input for postural control [71,93-98]. The sensory innervation of spinal ligaments is provided by Pacinian and Ruffini corpuscles, and free nerve endings [94,96-98]. Jiang et al [97] repeatedly stretched an intertransverse ligament of a young chicken. Tracing neuronal production of Fos protein through various sensory pathways, they identified afferent connections with the gracilis and cuneatus nuclei, the vestibular nuclei, and the thalamus. Yamada et al [96] identified a sympathetic innervation of the upper cervical posterior longitudinal ligament, from fibers projecting from the stellate ganglion. Interestingly, Sjolander et al [71] discuss how spinal ligamentous afferent information is at least partially responsible for mediating the reflex activity of its associated muscle spindles. They concluded that although muscle spindles may be dominant over ligament afferent input, maximal accuracy regarding joint position sense requires both sets of joint proprioception.

The cervical spine also contains an intricate muscular afferent network, given the numerous anterior and posterior cervical muscles. The upper cervical spine contains a higher density of muscle spindles than in any other spinal region [70]. Many authors have tested the function of cervical afferents by applying vibration to both normal subjects and those with specific neurological deficiencies [99-112]. For example, Ledin et al [106] found that vibratory stimulation of the calf muscle creates body sway in the sagittal plane, and this sway is significantly altered by flexion or extension, but not rotation, of the head. They suggest that either altered neck muscle position or utricle and saccule proprioceptive interaction may account for this functional deficit during vibratory stimulation. Sagittal postural sway was also observed when vibration was applied to the lower posterior cervical musculature [102]. Like the vestibular apparatus, Ivanenko et al [102] suggest that postural afferents from the cervical muscles are also processed within the parameters of the visual field. In another lower leg vibration study by Vuillerme et al [112], they found that vibration applied to the lower leg in upright humans also increased postural sway, as did muscular fatigue in the lower leg. However, when vibration was applied to a fatigued muscle, the postural sway did not increase as the authors had hypothesized. The authors suggest that the central nervous system effectively disregards the afferent information provided by a fatigued muscle, thus relying on other postural control mechanisms, such as the visual and vestibular systems, to provide this lost control [112]. When vibration is applied to upper cervical musculature, a greater degree of postural compensation occurs compared to that occurring from lower cervical vibration, suggesting that the upper cervical spine has an even greater role in posture regulation through visual orientation, than even the lower cervical spine. This observation is supported by previous works from Bogduk [113-117] showing how injury or pathology of the upper cervical spine produces a significant amount of noxious afferent input into the central nervous system, which may interfere with postural control. This is apparent in individuals with previous neck trauma and concurrent chronic neck pain [118-120].

Vibrational studies have also been conducted on individuals with certain postural deficiencies. In a study comparing normal subjects to those with labyrinthine deficiency, Popov et al [109] observed that vestibular-deficient subjects could not achieve the same ocular tracking of a fixated target image as matched controls. The authors conclude that this may result from changes in the cervico-ocular reflex, which will be discussed later in this review. Interestingly, a study by Karnath et al [103] demonstrated that the head tilt associated with spasmodic torticollis can be significantly reduced at least temporarily, when subjected to cervical vibration for 15 minutes. This finding led the authors to conclude that the muscular spasm associated with spasmodic torticollis may be the result of aberrant afferent input relaying head position to the central nervous system. Bove et al [100] demonstrated how asymmetrical vibration of the sternocleidomastoid affects locomotion. They found that subjects would rotate away from the side of vibration when applied during stepping. However, when the vibration was applied before stepping, compensatory rotation occurred opposite the initial rotation. The authors suggest that cervical input plays a major role during locomotion, and a lesser-coordinated role during static posture. Two other studies by Strupp et al [111] and Betts et al [99] also demonstrated the ability of the cervical afferent input to compensate for a decline in vestibular function.

### Visual and Cervical Interactions

While the visual field and the vestibular apparatus have intimate connections for postural control, they also have well-known connections with the cervical spine. Arising from sensory receptors in the cervical spine are three well-known reflexes that aid in postural control: 1) the cervico-ocular reflex [121-124], 2) the cervicocollic reflex [124-128], and 3) the vestibulocollic reflex [128-142], which will be discussed later in this review.

The cervico-ocular reflex serves to orient eye movement to changes in neck and trunk position [143-148] Similarly to other postural reflexes, a basic understanding of the cervico-ocular reflex is achieved by studying patients with specific postural reflex deficiencies. For example, Chambers et al [143] tested 6 patients with bilateral vestibular loss, and 10 controls. They found that light pattern stimulation caused at least a marginal amount of increased cervico-ocular reflex response, which was compensatory in half of the subjects. The authors concluded that the cervico-ocular reflex may at least partially compensate for absent vestibular function and vestibulo-ocular reflex. In another study by Bronstein and Hood [144], the postural control role of the cervico-ocular reflex was also tested in 12 patients with absent vestibular function. They found that the cervico-ocular reflex in patients with absent vestibular function seems to take on the lost function of the vestibulo-ocular reflex during specific postural tasks, such as ocular tracking in the direction of a visual target. Heimbrand et al [145] also studied 5 patients with vestibular absence to identify the compensatory nature of the cervico-ocular reflex. Their findings demonstrate a high degree of plasticity in the cervico-ocular reflex. The authors found that the cervico-ocular reflex could be modified with the addition of optical lenses, where magnifying lenses increase cervico-ocular response. The use of reduced lenses decreased the response. They also found that afferent input from the trunk, cognitive interpretation, and both peripheral and foveal retinal information all contributed to the observed cervico-ocular reflex plasticity. This information seems to be important for cervico-ocular stabilization of the visual field in space and in relation to a stationary neck and movable trunk. In an earlier study by Bronstein et al [146], they found that when the absence of vestibular function was present concurrently with reduced optokinetic reflex or ocular rolling response, the plastic adaptation of the cervico-ocular reflex did not seem to compensate for the vestibular absence. This suggests a necessity of an intact optokinetic reflex for optimal cervico-ocular response.

While cervico-ocular responses have been repeatedly observed in vestibular deficient subjects, its importance in healthy human subjects is debatable [121,147]. Schubert et al [147], in a study of 3 patients with unilateral vestibular dysfunction, could not establish any evidence of a cervico-ocular response in any of the 7 controls or in 2 of the 3 patients. In the single patient with evidence of cervico-ocular response, a change in the reflex could only be obtained following 10 weeks of vestibular exercises. More specifically, however, the cervico-ocular reflex can be subdivided into a slow phase of the response and a quick phase [121]. Jurgerns and Mergner [121] found that while the slow phase of the cervico-ocular reflex has no functional significance in humans, the quick phase does contribute to ocular stabilization and orientation to changes in neck and trunk position, during certain postural tasks. The quick phase of the cervico-ocular reflex also appears to be significantly adaptable in a relatively short period of time. Rijkaart et al [148] tested 13 healthy adults by subjecting them to trunk rotation in a dark room, thus providing conflicting somatosensory and visual input to test the function of the cervico-ocular reflex. They found a significant amount of cervico-ocular adaptation could be achieved in as little as 10 minutes of constant visual and somatosensory input. This may have important clinical benefits, and will be discussed further in the second part of this review.

Perhaps more widely known for its role in postural control, the cervicocollic reflex serves to orient the position of the head and neck in relation to disturbed trunk posture [149]. This reflex, acting similarly to a stretch reflex [149], involves reflexive correction of cervical spine position through co-contraction of specific cervical muscles, including the biventer cervicis, splenius capitis and cervicis, rectus capitis posterior major, and the obliquus capitis inferior [127]. The cervicocollic reflex is activated in response to stimulation of muscle spindles located in these muscles. This reflex seems to modulate upright cervical posture in close communication with the vestibulocollic reflex [124,125], which will be discussed later. There also seems to be a significant amount of overlap in the pathways and functions of the cervicocollic and vestibulocollic reflexes, perhaps to readily compensate for injury or reduction in either of these two reflexes [126]. The vestibulocollic reflex seems more sensitive to changes in head position in the horizontal plane, while the cervicocollic reflex seems more sensitive to vertical plane positional changes [125]. Given the high density of muscle spindles in the cervical musculature, the cervicocollic reflex possesses a high degree of sensitivity to relatively small cervical stimuli. This suggests that this reflex may heavily rely upon muscle spindle afferents to provide postural information, so that immediate cervical postural corrections can be made [127]. Evidence of these immediate changes was illustrated by Keshner et al [125], where patients performed simultaneous postural and cognitive tasks with and without weight placed on top their heads. They found that adding weight to the head did not significantly change head or neck position, suggesting an immediate and compensatory response to the added weight.

### Vestibular and Cervical Interaction

Perhaps one of the most well studied postural reflexes; the vestibulocollic reflex maintains postural stability by actively stabilizing the head relative to space. It does this by reflexively contracting cervical muscles opposite of the direction of cervical spine perturbation [115,139]. In order to evaluate the mechanisms and efferent pathways of this reflex, several studies targeting this reflex using EMG recordings of various cervical muscles have been conducted [126,133,134,136,138]. The vestibulocollic reflex, from input originating in the semicircular canals, utricle, and saccule, stabilizes the head in space in response to even the slightest of head perturbations occurring in the horizontal plane [128,134,139,141,142]. From this perspective, the vestibulocollic reflex also acts much like a stretch reflex. Muscles that have been studied in connection with this reflex include the sternocleidomastoid [130,131,133,136], biventer cervicis, splenius cervicis and capitis, and the longus capitis [111].

There is an important distinction to make when discussing the vestibulocollic reflex. It should be noted that this reflex is distinct and largely dissociated from the vestibulospinal reflex, which orients the extremities to the position of the head and neck. Welgampola and Colebatch [138] found that the vestibulocollic reflex is not significantly affected by stimulation of lower extremity afferents, such as when a subject is placed in an upright posture on a narrow base and deprived of vision and external support. Likewise, Allum et al [128] showed that activation of the vestibulocollic reflex is mainly dependent upon stimulation of cervical afferents directly.

Another important aspect of the vestibulocollic reflex is the neural contribution it receives from the reticular formation [140,141]. This reticulospinal contribution is important because it may allow a "globalization" of this reflex, meaning that connection to the reticular formation allows postural information carried by the reflex pathway to be interpreted by several other central nervous system pathways, perhaps allowing the CNS as a whole to adapt to postural changes. These reticular connections also facilitate quicker vestibulocollic responses, and help increase the sensitivity of the vestibulocollic reflex to other postural afferents in related but divergent pathways [140].

### Neurological Development of Postural Control

Any discussion pertaining to the mechanisms through which postural adaptations are made must include information on the development of these postural adaptive mechanisms. As already suggested, the visual field may be the most heavily favored of the postural reflexes. As many authors have pointed out, an infant's orientation to the extrauterine environment is dictated almost exclusively by the visual field [150-153]. As Precht [152] discussed, a human newborn is poorly adapted to the gravitational environment, given poor muscle power and weak or absent reflex control of the head and trunk. Infants at 2 months of age begin to consistently rely upon visual cues to orient the head and body. At 4 to 6 months, as infants begin to crawl, other postural reflexes begin to play important roles, such as joint mechanoreceptors and the vestibular system [154]. Pope [153] showed that as infants begin to crawl, reliance on visual feedback is reduced. Perhaps not coincidentally, however, certain stages of upright postural progression may be characterized by periods of reliance on the visual system as the primary mechanism of postural regulation. For example, Butterworth and Hicks [151] pointed out that visual feedback is again favored as the infant masters motor control of the trunk and starts to sit upright independently. Lee and Aronson [155] observed a similar pattern of visual predominance as the infant begins to stand.

From this material, it is logical to conclude that as a child is born, concerning progression from crawling, to sitting upright, to standing, reflexive head control seems to be the primary factor necessary for upright postural control. This sequence of postural development is predicated upon mastering reflex control of head position relative to gravity so that trunk and lower extremity control can be learned using a fixed reference point. This conclusion is further supported by evidence that after reflex control of the head is learned, standing requires a coordinated response of the lower extremity musculature to balance the position of the head over the base of support. As Woollacott et al observed [154], neuromuscular responses of the lower extremity are coordinated much earlier than trunk and upper extremity muscles. The neuromotor responses of the pelvic girdle and lower extremity are collectively termed the pelvo-ocular reflex [156], which serves to orient the body region in response to head position and anticipatory visual reference cues. The significance of this reflex may be attributed to the early development of hip and leg coordination. Neuromuscular coordination of the trunk may not be fully developed until the child reaches 7–10 years of age [154].

Although visual input for postural control seems to predominate in early life, they may be some explanation as to why this occurs. Because the visual system functions independent of gravity [157], this system is not affected by gravitoinertial changes. Therefore, it can provide the most consistent reference point from which to orient the head and neck. Additionally, previous studies have demonstrated that infants cannot process and integrate postural input from multiple sources, such as from joint mechanoreceptors and the vestibular system. In a study of 4–6 month old infants, Woollacott et al [154] found that infants using both visual and vestibular cues were able to correctly orient to a moving platform 60% of the time. However, when the infants were blindfolded using goggles, their postural responses were correctly oriented 100% of the time, suggesting an inability of the infant to process two different sources of postural stimulus simultaneously. By 8–14 months, however, infants appeared to consistently adapt to postural stimuli from both sources of sensory input.

### Biomechanical Development of Postural Control

Aside from the neurological development of postural control, it is important to discuss the biomechanical development of postural control, especially as it relates to the spine. Since the spine is the literal backbone of upright postural support, structural and functional development of the spine also appear to be consequences of upright adaptation to a gravitational environment. The sagittal curves of the spine allow for a balance between strength and flexibility, while also resisting the axial compressive force of gravity [158]. These sagittal curves are not fully developed at birth. Rather, they are formed as a consequence of adaptation to the external environment (gravity). *In utero*, the fetal spine is shaped more as a C-shaped curve. This shape is more suited to adapt to a microgravitational environment. However, as the fetus grows and occupies more of the uterus, much of the watery environment is lost. Therefore, the fetal spine begins to adapt and take on a structure more suited for gravitational adaptation. Bagnall et al [159] suggested that the cervical curve is fully developed in utero. However, their study used postmortem fetuses artificially positioned and radiographed, although the authors note that much attention was given to replicating the fetal position in the uterus. Although they note no visual abnormalities, no information is given as to the cause of fetal demise or maternal history. Therefore, it is possible that these fetuses are not representative of the average healthy fetal population. Panattoni and Todros [160] demonstrated through ultrasonography that both the cervical and lumbar curves are visually developed by the 24^th^–26^th ^week of gestation. This may be due to the morphological development of the cervical facet joints and discs.

**Figure 1:**
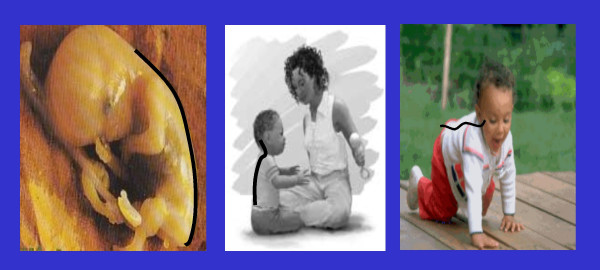
This figure illustrates the development of the sagittal spinal curves. In the womb, the fetal spine is more of a C-shape (left). As the child begins to hold his head up, the cervical curve is developed and reinforced (middle). Finally, as the child begins to crawl, gravity helps to develop the lumbar curve, a requisite for a bipedal upright stance (right).

**Figure 2:**
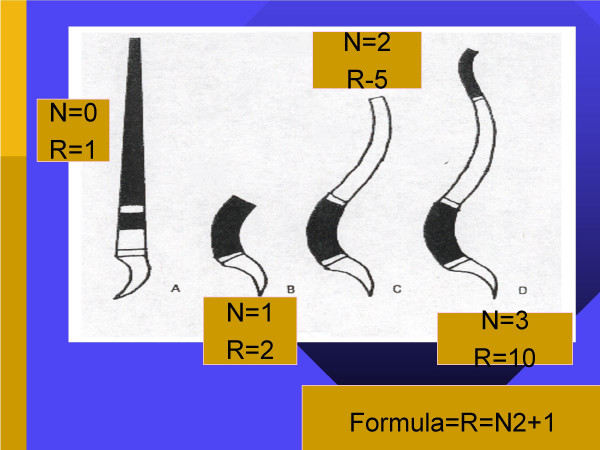
The resistance (**R**) of any curved column to compression forces is directly proportional to the square of the number of curves (**N**) plus one (**R **= **N**^2 ^+ 1). Therefore, the fetal spine with its single curve has a resistance value of 2 (1^2 ^+ 1 = 2). This is not enough to resist the forces of gravity against the head, neck, and upper trunk as we saw in Figure 1. The development of the cervical curve increases the resistance value of the spine by 2.5 times (2^2 ^+ 1 = 5).

The extrauterine environment changes the compressive force and force vectors upon the spine. As previously mentioned, the newborn muscle strength is not sufficient to maintain upright head posture. From a mechanical standpoint, creating more of a mechanical advantage can compensate for this lack of muscle strength. The C-shaped spine provides two intrinsic lever arms from which the paraspinal muscles attach and initiate movement. However, since the spinal muscles are too weak to maintain upright head position, shorter lever arms must be developed to overcome this muscular deficit. The forward cervical curve creates two more functional lever arms, giving the cervical spine muscles the mechanical advantage necessary for upright head stabilization and movement. Figure 1 depicts these developmental stages. As the child begins to crawl, the lumbar curve is developed as a result of the downward pull of gravity. Once the lumbar curve is developed, two more lever arms are created, providing the lumbopelvic musculature with the leverage necessary to allow an upright standing posture. From an engineering perspective, the resistance of a curved column is directly proportional to the square of the number of curves plus one [158]. Therefore, a C-shaped fetal spine contains two lever arms, for a resistance of 5 (2 × 2 + 1 = 5). When the child develops a cervical curve, two additional lever arms are created, thereby increasing the resistance to 17 (4 × 4 + 1 = 17). Finally, the lumbar curve development further increases the resistance of the spine to 37. An illustration of this is shown in Figure 2. The creation of these lever arms allows the spinal muscles to maintain upright posture more efficiently. Initially, upright postural control is a voluntary muscular task. However, as the spinal muscles are repeatedly required to perform these tasks, the tasks become automated. As a nerve impulse passes through a set of neurons at the exclusion of others, it will take the same course on future occasions and each time it traverses this path the resistance will be smaller [161,162]. As these postural neuromotor pathways are facilitated, they become the basis for the neurophysiologic reflexes governing involuntary postural regulation. Harbst [163] previously reported that repeated voluntary performance of a postural task becomes faster and easier to perform as neuromotor pathways are reinforced. As the infant begins to hold his/her head upright, the postural muscles required to perform that task are activated. As this task is repeated, holding the head upright becomes an involuntary, automated process under the direction and control of the postural reflexes via the facilitated neuromotor pathways. The same neurological learning processes are invoked as the child begins attempting to stand upright. At this point, postural muscles are required to perform many functions simultaneously. Joints of the thoracic and lumbopelvic spine, as well as the hips, knees and ankles, must all be actively stabilized by the surrounding regional musculature. Meanwhile, the global spine and posture must balance the position and weight of the head, neck and trunk above their base of support, the feet. This active muscular stabilization also increases the stress on musculotendinous junctions and osseous attachments, increasing the rate at which the skeletal frame ossifies. As the process of standing is repeated, neuromotor control of the lower extremity and spinal muscles is coordinated with the postural reflexes of the head and neck through cerebellar integration, thereby developing a cohesive network of involuntary postural control.

### Reflex Hierarchy

Human upright posture is developed and maintained in response to earth's gravity. Perhaps the best way to study the effects of gravity upon the human spine and nervous system is to study humans as they actively adapt to environments where the gravitational field is altered or absent. Clues to reflex hierarchy and reference may be determined as a consequence of forced adaptation to a new external environment.

Since space travel has become a reality, several studies have demonstrated the effects of microgravity on human posture. Perhaps most importantly, it would appear that a postural reflex hierarchy may exist irrespective of the external environment. For example, a study by Baroni et al [164] evaluated two astronauts during space flight using kinematic analysis. The astronauts were instructed to perform specific axial movements from an erect, upright posture. Their postures and movements were recorded before, during, and after the movement performance. The authors found a pronounced forward trunk lean when the eyes were closed compared to eyes open. They suggest that visual input for postural control may be independent of gravity-based postural cues. This conclusion is also supported by research from other authors. Koga [157] studied the eye movements of humans during spaceflight. He found that purposeful eye movements showed similar accuracy of target fixation and saccade compared to pre-flight eye movements. Further, Koga [157] reported that neck muscle activity was not coordinated with ocular movement during spaceflight, although oculocervical coordination was observed under Earth's gravity. These findings demonstrate a visual preference for postural control in altered external environments, and that cervical spine afferents are gravity dependent. More specifically, extraocular muscular afferents are highly coordinated and function independently of gravitational changes. Regardless of the gravitational environment, visual afferents and cues provide an external reference for maintaining upright posture, even when somatosensory afferents and internal references are absent or conflicting [165-167].

In the presence of Earth's gravity, the vestibular system plays a major role in monitoring changes in head position, primarily through the utricle and saccule [168]. However, initial exposure to microgravitational environments reduces the effects of the vestibular organs on posture regulation. Clarke et al [168] showed that vestibular control of posture recovers only after prolonged exposure to microgravity. They suggest that cervical spine afferents may play a role in vestibular recovery.

### Postural Body Scheme

Another method for observing and documenting the interactions of postural reflexes is to study the causes and factors associated with space motion sickness. This sickness is simply a result of conflicting postural input into the central nervous system. This sickness is common in the first days of spaceflight, and resolves as adaptation to microgravity occurs [169]. The occurrence of space motion sickness provides a framework from which postural control theories attempt to explain upright posture regulation in direct response to gravity.

A conceptual model called the postural body scheme [165,170-173] represents the internal reference point by which upright posture is regulated. Vertical body orientation, corrective postural reactions, and anticipatory postural adjustments are all organized based on this internal representation [170]. This postural body scheme remains stable during gravitational changes, even when mechanoreceptive and vestibular inputs are significantly decreased [174]. During periods of microgravity, space motion sickness is attributed to a conflict between visual postural inputs and afferents from the vestibular and somatosensory systems. The postural body scheme is centered around gravity acting as the vertical axis of space while in earth's gravity. However, this vertical axis is not present in microgravity, effectively eliminating the external reference point for many of the postural reflexes [175]. To observe these effects, Takahashi et al [176] performed Coriolis stimulation on five healthy subjects before and during space flight. The subjects were instructed to tilt their heads at varying speeds in both gravitational environments. Observations were recorded regarding eye movement, body sway, and motion sickness. They found that nystagmus was present under both conditions, although its duration was shorter in microgravity. However, body sway and sickness was not observed in microgravity, although they were apparent in normal gravity. Their findings provide evidence that visual control of posture is defined by an internal reference frame within the brain, not subject to changes in external environment. In an experiment conducted by Amblard et al [177], they recorded movements associated with head stabilization in two subjects during space flight. Their results also suggest a postural reflex hierarchy, with visual input, vestibular input, and postural body scheme among the most important. The main underlying commonality among all of these cited studies is the predominance of visual input and afferents in regulating upright postural control despite changes in the external gravitational environment. Finally, although the postural body scheme is an internal reference point for postural control, it may receive much of its information from visual input. Yakushin et al [178] performed vestibular stimulation on five subjects while their heads were immobilized. The subjects were then placed in a side lying position. Three-dimensional ocular movements were recorded during vestibular stimulation. The authors found that adaptations of the vestibulo-ocular reflex are gravity-dependent, and appear to be stored as a sort of short-term posture memory. They suggest that the vestibular nuclei may be responsible for the storage of this gravity-dependent posture information, since these nuclei form direct connections between vestibular and visual afferents.

Not only does the postural body scheme provide an internal framework for maintaining upright posture, but it also serves as a stable internal representation of biomechanical properties to guide and organize anticipatory postural adjustments and voluntary motor movements [179,180]. Understanding the conceptual model of the postural body scheme can be clinically beneficial to manipulative medical clinicians in that biomechanical functional improvement may ultimately rely on the patient's ability to learn novel neuromotor strategies for upright posture and gait.

## Discussion

This review has focused mainly upon the postural reflexes associated with the cervical spine and its constituent parts, and the special senses, specifically the eyes and inner ear. Obviously, there are other postural reflexes we did not cover in this review, including skin and surface receptors in the extremities [181,182], somatic graviceptors [183-185] located within the viscera, and baroreceptors located within the circulatory system [186,187]. However, they were not covered in this review for specific reasons. Skin and surface receptors in the lower extremity have been well illustrated in the chiropractic literature, especially in regards to postural control. Various authors have already shown improvements in postural control, via balance testing, using molded foot orthotics [188,189], for example. Given the extensive information already published [181,182] on this aspect of postural control, we did not address it here. However, this omission does not diminish its importance for postural control. Additionally, while somatic graviceptors and vascular baroreceptors also maintain certain postural regulatory functions, these components are not readily modifiable by manual medical methods. Our review has focused upon those postural reflexes that can be predicted and recruited by clinicians in chiropractic, physical therapy, physical medicine, and osteopathic medicine to ultimately help execute specific postural rehabilitation programs.

We attempted to review and discuss the anatomy and interactions of the various postural reflexes. However, with the amount of overlap found in many neurological processes, it is likely impossible to identify and outline each and every postural reflexive behavior. Also, as biomedical technology and research uncovers new areas of neurobiology and neurophysiology, we will no doubt find our present review of postural reflexes to be inadequate. The complexity of the nervous system has probably not yet been fully appreciated by medical and allied health practitioners, nor are its connections to all other physiological processes, including posture, fully documented and understood.

This review is important in that much of this information has largely been ignored in chiropractic and manual medicine. Although posture correction has gained significant popularity more recently, the neurological control of posture has been largely omitted. While previous reviews have outlined potential pathogenetic biomechanical configurations of the spine and spinal cord [18-20], neurophysiologic adaptation to normal and abnormal posture has not been extensively detailed. Given both the historical and clinical importance of the nervous system to overall health and well being [2,10], its involvement in something as important as postural control should be emphasized in future chiropractic literature.

Traditional chiropractic principles maintain that the nervous system is responsible for coordinating all other body systems [190]. Typically speaking, this perspective is applied to the other specific body systems, such as the cardiovascular, digestive, endocrine, and respiratory systems. However, in this paper, we detail the mechanisms and adaptive processes by which the nervous system also controls and coordinates our upright posture. In addition to regulating our internal environment, our nervous system, through its various postural reflexes, observes, analyzes, predicts, and adapts to changes in our external environment. Primarily, this external environment is gravity.

Chiropractors aim to evaluate and treat articular dysfunction of the spine to restore function, reduce pain, and encourage normal nervous system "integrity." [191] This has typically been performed by trying to determine spinal segmental alterations in alignment in relation to the vertebral segments above and below. However, from the data presented here, we suggest that the spine, as a singularly functioning entity, is subservient to the reflex adaptations made by the nervous system in relation to gravity. So, it may be worth trying to identify potentially putative postural reflex function(s). A theoretical example may be a thoracolumbar pain caused by a swayback posture. It is postulated that the swayback posture may be the result of a forward head posture relative to the trunk, thus causing a forward shift of the pelvic complex. This forward pelvic shift is mediated by the pelvo-ocular reflex [156], as outlined in our previous review. However, the underlying cause of this theoretical thoracolumbar pain and dysfunction may be the forward head posture forcing a compensatory swayback posture.

There are a couple of specific points made in this paper that we wish to highlight and relate to clinical practice, since these concepts are not currently explored or discussed in the chiropractic literature. First, as discussed earlier in this paper, we reviewed previous work by Bove et al [100] testing the cervical spine contribution to locomotion and static posture through unilateral sternocleidomastoid vibration. Again, their findings suggest that the cervical spine plays a larger role in locomotion and a smaller role in static upright posture. This information has important clinical implications that manual practitioners should consider. Locating specific postural reflex deficits may be achieved by subjecting patients to various postural and locomotive balance tasks to identify which function is being compromised. Although unproven, isolation and treatment of the specific deficient postural reflex may well mean the difference between treatment success and failure.

Two separate studies by Karnath et al [103] and Vuillerme et al [112] compared the afferentation of a fully active muscle and a fatigued muscle. The collective results of these studies may have very important clinical relevance. If fatigued muscles are not able to transmit somatosensory information to the central nervous system, then upright postural control may be compromised if maintaining a given static posture requires a large amount of constant isometric muscular contraction. Certain subpopulations may therefore be advised to undergo posture correction, such as those elderly who are at risk for balance failures and/or hip fractures. These balance failures may be at least partially attributed to lack of somatosensory input from fatigued postural muscles. This hypothesis is certainly worthy of research.

While neurological disturbances have been well documented in other contexts, such as whiplash-associated disorders [76,89,90,92,192], neurological disturbances resulting from chronic abnormal posture have not been elucidated. This may be due to a more narrow focus upon only the mechanical components of the spine and posture [16,17,22-27]. We hope that this review will help to shed some light upon the postural adaptations and responses that may not only *cause *neurophysiological dysfunction, but also those that may occur *because *of it.

## Conclusion

Upright human posture is maintained reflexively by a vast network of peripheral and central nervous pathways designed to provide instantaneous input regarding both internal and external environmental factors. In this review, we outlined those postural reflexes related to pathways and structures involving the cervical spine, the eyes, and the inner ear. How these structures and pathways obtain somatosensory input, interact with each other, and modulate postural changes and corrections has been described here. While there are many other postural control mechanisms we did not discuss in this review, we chose to outline those reflexes that may be of primary importance to practitioners within the manual healing arts. This review may shed some light upon the idea that vertebral misalignments or fixations are not random injury- or activity-induced events. Rather, they may be a consequence of an adaptive postural process to the external environment mediated by the nervous system through its extensive network of postural reflexes. Research into this concept is necessary before clinical utility is determined.
